# MicroRNA profiling in ischemic injury of the gracilis muscle in rats

**DOI:** 10.1186/1471-2474-11-123

**Published:** 2010-06-17

**Authors:** Ching-Hua Hsieh, Jonathan Chris Jeng, Seng-Feng Jeng, Chia-Jung Wu, Tsu-Hsiang Lu, Po-Chou Liliang, Cheng-Shyuan Rau, Yi-Chun Chen, Chia-Jung Lin

**Affiliations:** 1 Department of Plastic and Reconstructive Surgery, Chang Gung Memorial Hospital, Kaohsiung Medical Center, Chang Gung University College of Medicine, Taiwan; 2 Business BA at University of Texas at Dallas, 800 W Campbell Road, Richardson, TX 75080, USA; 3 Department of Neurosurgery, E-Da Hospital, I-Shou University, Kaohsiung, Taiwan; 4 Department of Neurosurgery, Chang Gung Memorial Hospital, Kaohsiung Medical Center, Chang Gung University College of Medicine, Taiwan

## Abstract

**Background:**

To profile the expression of microRNAs (miRNAs) and their potential target genes in the gracilis muscles following ischemic injury in rats by monitoring miRNA and mRNA expression on a genome-wide basis.

**Methods:**

Following 4 h of ischemia and subsequent reperfusion for 4 h of the gracilis muscles, the specimens were analyzed with an Agilent rat miRNA array to detect the expressed miRNAs in the experimental muscles compared to those from the sham-operated controls. Their expressions were subsequently quantified by real-time reverse transcription polymerase chain reaction (real-time RT-PCR) to determine their expression pattern after different durations of ischemia and reperfusion. In addition, the expression of the mRNA in the muscle specimens after 4 h of ischemia and reperfusion for 1, 3, 7, and 14 d were detected with the Agilent Whole Rat Genome 4 × 44 k oligo microarray. A combined approach using a computational prediction algorithm that included miRanda, PicTar, TargetScanS, MirTarget2, RNAhybrid, and the whole genome microarray experiment was performed by monitoring the mRNA:miRNA association to identify potential target genes.

**Results:**

Three miRNAs (miR-21, miR-200c, and miR-205) of 350 tested rat miRNAs were found to have an increased expression in the miRNA array. Real-time RT-PCR demonstrated that, with 2-fold increase after 4 h of ischemia, a maximum 24-fold increase at 7 d, and a 7.5-fold increase at 14 d after reperfusion, only the miR-21, but not the miR-200c or miR-205 was upregulated throughout the experimental time. In monitoring the target genes of miR-21 in the expression array at 1, 3, 7, 14 d after reperfusion, with persistent expression throughout the experiment, we detected the same 4 persistently downregulated target genes (*Nqo1*, *Pdpn*, *CXCL3*, and *Rad23b*) with the prediction algorithms miRanda and RNAhybrid, but no target gene was revealed with PicTar, TargetScanS, and MirTarget2.

**Conclusions:**

This study revealed 3 upregulated miRNAs in the gracilis muscle following ischemic injury and identified 4 potential target genes of miR-21 by examining miRNAs and mRNAs expression patterns in a time-course fashion using a combined approach with prediction algorithms and a whole genome expression array experiment.

## Background

Skeletal muscle ischemia is an important clinical problem that may result in a significant high rate of morbidity and mortality. Despite extensive experimental work that is directed toward the treatment and prevention of established ischemic injuries, the clinical outcome has not appreciably changed over the past decades [1]. This may be related to the fact that the pathophysiology of this complex event is still incompletely understood. The microRNAs (miRNAs) are a novel regulatory class of noncoding, single-stranded RNAs of approximately 22 nucleotides, which have recently been identified to play critical roles in normal development and physiology, as well as in disease development [2,3]. The discovery of miRNAs has broadened the overall understanding of the mechanisms that regulate gene expression, with the addition of an entirely novel level of regulatory control. Both basic and clinical studies suggest that miRNAs are important regulators of cell differentiation, growth, proliferation, and apoptosis [4-6]. Estimates indicate that miRNAs may regulate up to one-third of the mammalian genome [7]. However, each miRNA possibly targets many different mRNAs and the same target gene may be regulated by a given miRNA in different situations, allowing for enormous complexity and flexibility in their regulatory potential [7-9]. Therefore, although a large number of miRNAs have been discovered, only a few target genes have been identified and the functions of most of them remain unknown.

The miRNAs repress protein expression at the posttranscriptional level, mostly through base pairing to the 3' untranslated region (UTR) of the target mRNA, thus leading to its degradation and/or reduced translation. Earlier, miRNAs were thought to primarily repress their target genes at the protein level without affecting mRNA stability [10,11]; however, increasing evidence indicates that miRNAs silence genes by multiple mechanisms, including the degradation of their target mRNAs [12,13]. For the more highly repressed targets, mRNA destabilization usually constituted the major component of repression [14], thus making the investigation of the mRNA:miRNA association by monitoring miRNA and mRNA expression on a genome-wide basis a novel analytical approach to understand the miRNA-mediated regulation [15,16].

Dysregulated miRNA expression has been reported to be involved in the transient focal ischemic brain [16,17] and in the ischemia-reperfusion injury of the heart [18,19]. In addition, the endogenously synthesized miRNAs demonstrated to be cardioprotective following ischemia-reperfusion injury [19]. Moreover, there is increasing evidence for the involvement of microRNA in myopathies [20-22]; a number of microRNAs have been characterized as regulators of skeletal muscle development and diseases [23,24] as well as of skeletal muscle remodeling [25]. Given the importance of miRNAs in the pathophysiology of the muscle, we hypothesized that microRNAs could be involved in the skeletal muscle in response to ischemic injury. First, we addressed this hypothesis using a microarray-based screening. The expression profile was subsequently verified with real-time reverse transcription polymerase chain reaction (real-time RT-PCR). Potential target genes were identified by monitoring the miRNA and mRNA expression on a genome-wide basis.

## Methods

### Animal surgery and tissue preparation

The experiments were performed on adult male Sprague-Dawley rats weighing 250-300 g. The rats were randomly assigned to the sham-operated control group and the ischemic group. The rats were anesthetized by an intraperitoneal injection of 500 mg/kg chloral hydrate and prepared in the prone position. The gracilis muscle flap was dissected under the microscope by the same surgeon. The flap was isolated on its dominant and minor vascular pedicles. We used a standard microvascular technique for the sham-operated control group. The minor vascular pedicle was electrocauterized and transected. Ischemia was induced in the ischemic group by placing a microvascular clamp carefully across the proximal dominant vascular pedicle to the gracilis muscle. The gracilis muscle was allowed to perfuse in the sham-operated control group. After the indicated ischemic time (0.5, 1, 2, and 4 h), the microvascular clamp was removed. Good vascular inflow and outflow through the pedicle was verified under direct magnified vision. The incision wound was closed with interrupted sutures (4-0 nylon) and the animals were allowed to awaken in the remaining periods of reperfusion. For the miRNA array experiments, gracilis muscles after 4 h of ischemia and 4 h of reperfusion were used in 2 replicate experiments. First, we performed evaluation experiments to determine the minimal ischemic time that would induce miRNA expression before we investigated the miRNA expression in the real-time RT-PCR experiments. For that, the muscle specimens were either harvested after 4 h of ischemia and reperfusion for 0, 2, 4, 8, and 24 h and for 3, 7, and 14 d or the ischemia times were set at 0.5, 1, and 2 h with perfusion for 4 h. We used the muscle specimens that underwent ischemic injury at 1, 3, 7, and 14 d after reperfusion for the whole genome expression experiments. The harvested muscles were frozen in isopentane chilled in liquid nitrogen and stored at -80°C. All housing, surgical procedures, analgesia, and assessments were performed in accordance with the Animal Care Guidelines and were approved by the Animal Care Committee at the Chang Gung Memorial Hospital.

### RNA isolation

Total RNA was extracted using the mirVana miRNA Isolation kit (Ambion, Austin, TX, USA). The purified RNA was quantified by determining the absorbance at 260 nm using an SSP-3000 Nanodrop spectrophotometer (Infinigen Biotech, City of Industry, CA, USA). The quality of the purified RNA was evaluated on a Bioanalyzer 2100 (Agilent Technologies, Palo Alto, CA, USA) for the miRNA array and whole genome expression analyses.

### Expression of miRNAs

A rat miRNA array (G4473A, Agilent Technology) which includes 350 rat miRNAs (Sanger miRBase Release 10.1) was used to identify the upregulated miRNAs in the gracilis muscles after 4 h of ischemia and 4 h of reperfusion, with total four one-color miRNA arrays for two sham-operated and two experimental muscle specimens. One hundred nanogram of total RNA was dephosphorylated for 30 min at 37°C with 11.2 units calf intestine alkaline phosphatase (GE Healthcare Life Sciences, Uppsala, Sweden). The reaction was terminated by heating at 100°C for 5 min and immediate cooling to 0°C. DMSO (5 μl) was then added. The solution was heated to 100°C for 5 min and immediately cooled to 0°C. Ligase buffer and BSA were added and ligation was performed by adding pCp-Cy3 (50 μM) and 15 units T4 RNA ligase in 28 μl. The mixture was incubated at 16°C for 2 h. The labeled miRNAs were desalted with MicroBioSpin6 columns (BioRad, Hercules, CA, USA). Subsequently, 2× hybridization buffer was added to the labeled mixture to a final volume of 45 μl. The mixture was heated for 5 min at 100°C and immediately cooled to 0°C. Each 45-μl sample was hybridized onto an miRNA array at 55°C for 20 h. After hybridization, the slides were washed at room temperature for 5 min in Gene Expression Wash Buffer 1 and then for 5 min in Gene Expression Wash Buffer 2. The slides were scanned on an Agilent microarray scanner G2565A. The sensitivity settings were 100% and 5%. Agilent Feature Extraction software version 9.5.3 was used for image analysis. The microarray data were analyzed using GeneSpring GX 7.3.1 (Agilent Technologies). The miRNA expression was considered significantly different when values in the muscles of the experimental rats were more than double of those of the sham-operated controls in 2 replicate experiments. ANOVA was employed to compare the average values of the miRNA probes in all samples and yielded significant *P *values (<0.05) in all cases.

### Quantification of miRNAs expression

Those upregulated miRNAs (miR-21, miR-200c, and miR-205) that were identified from the miRNA array were quantified by real-time RT-PCR with the Applied Biosystems 7500 (Applied Biosystems, USA). We isolated total RNA from the harvested muscle with the mirVana miRNA Isolation Kit (Ambion, USA) as well as a TaqMan miRNA Assay kit (Applied Biosystems, USA) according to the manufacturers' instructions. The thermal cycling conditions comprised an initial denaturation step at 95°C for 10 min and 40 cycles at 95°C for 15 s and 60°C for 1 min. Each value of miRNA expression was represented relative to the expression of small RNA 4.5 S, which was used as an internal control. The fold-expression of induction was calculated as the relative expression values obtained in each condition in conjunction with the standard deviation compared with the relative expression values from the gracilis muscles of the sham-operated control group. The comparison between the groups included ANOVA and the appropriate posthoc test to compensate for multiple comparisons (SigmaStat, San Rafael, CA, USA). P-values of less than 0.05 were considered significant.

### Whole Genome Microarray Analyses

Four two-color Whole Rat Genome 4 × 44 k oligo microarrays (Agilent Technologies) were used to detect the change of the transcripts in the experimental gracilis muscles. Expression levels of the mRNAs in the gracilis muscles after 4 h of ischemia and 1, 3, 7, and 14 d of reperfusion with 1 specimen at each of the abovementioned time points were detected with the oligo microarray and compared against those expression levels of the sham-operated control specimens at the same indicated time. The microarray experiments were carried out according to the manufacturer's protocols. In brief, 0.5 μg of total RNA was amplified using a Fluorescent Linear Amplification Kit (Agilent Technologies) and labeled with Cy3-CTP or Cy5-CTP (CyDye, PerkinElmer, CA, USA) during the *in vitro *transcription process. The RNA from the experimental muscle was labeled with Cy5 and the RNA from the sham-operated control muscle RNA was labeled with Cy3. Then, 0.825 μg of the Cy-labeled cRNA was fragmented to an average size of about 50-100 nucleotides by incubation with the fragmentation buffer (Agilent Technologies) at 60°C for 30 min. Subsequently, the fragmented labeled cRNA was pooled and hybridized to the Whole Rat Genome microarray at 60°C for 17 h. After washing and drying with a nitrogen gun, the microarrays were scanned with an Agilent microarray scanner (Agilent Technologies) at 535 nm for Cy3 and 625 nm for Cy5. The scanned images were analyzed with the Feature Extraction software 9.5.3 (Agilent Technologies). We used image analysis and normalization software to quantify the signal and background intensity for each feature and to substantially normalize the data by the rank-consistency-filtering LOWESS method. The differentially expressed mRNAs were selected if there was a 2-fold change between the experimental muscles and the sham-operated control muscles.

### Prediction of the potential target genes of miRNAs

To date, there is not 1 algorithm that outperforms others in terms of sensitivity and specificity. We identified the potential targets of those upregulated miRNAs by combined analysis of the downregulated mRNAs in the whole genome expression microarray and the commonly used web tools for bioinformatics algorithms, including 3 of the most used prediction websites: miRanda http://www.microrna.org/microrna/home.do, PicTar http://pictar.mdc-berlin.de/, and TargetScanS http://www.targetscan.org/, as well as 2 additional algorithms, MirTarget2 http://mirdb.org/miRDB/ and RNAhybrid http://bibiserv.techfak.uni-bielefeld.de/rnahybrid/. The *in silico *predicted targets genes were compared to the list of 2-fold downregulated mRNA transcripts (derived from the whole rat genome microarray experiments) by examining miRNAs:mRNAs expression pairs in a time-course fashion. The genes that were identified in both methods were considered as potential target genes regulated by a given miRNA.

## Results

### Expression profile of the miRNAs

**Figure 1:**
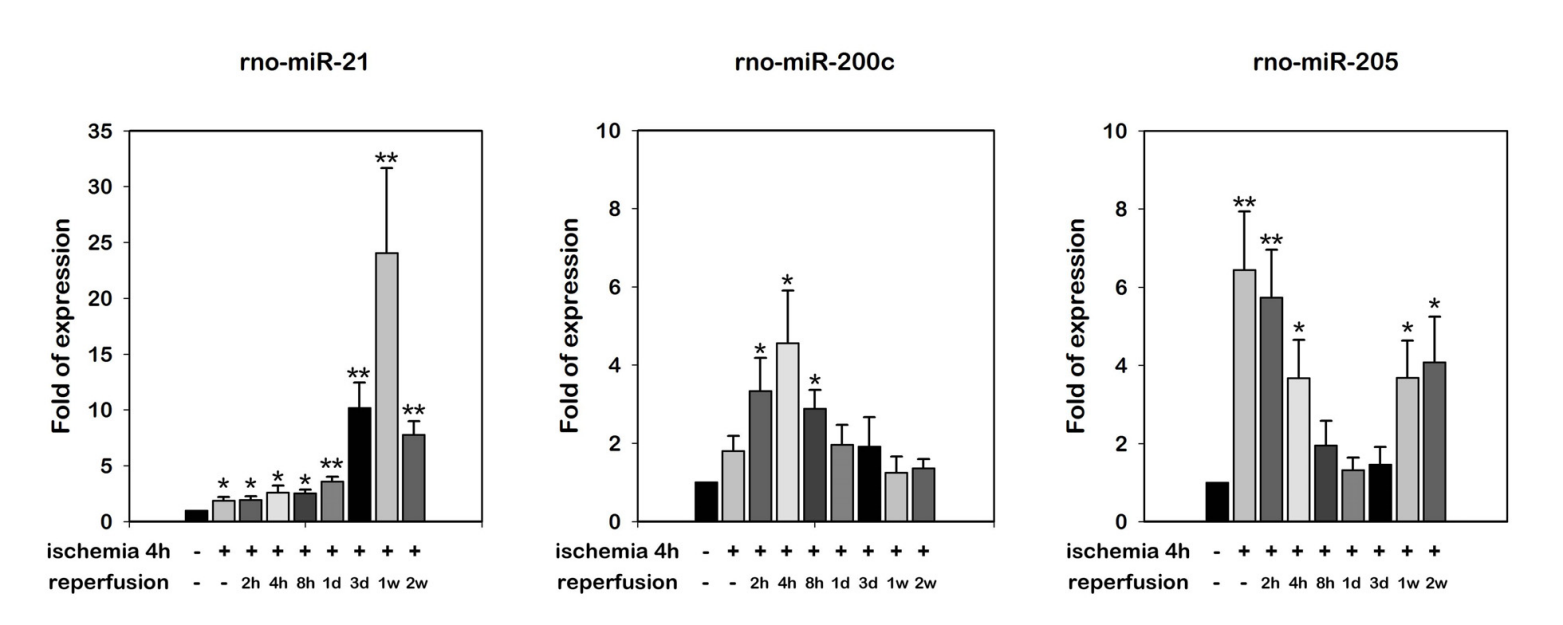
**Expression of miR-21, miR-200c, and miR-205 detected with real-time RT-PCR in the gracilis muscles following 4 h of ischemia and reperfusion for indicated times**. Bars represent means ± standard deviation of 6 independent experiments; *, P < 0.05 vs. sham-operated control.

**Figure 2:**
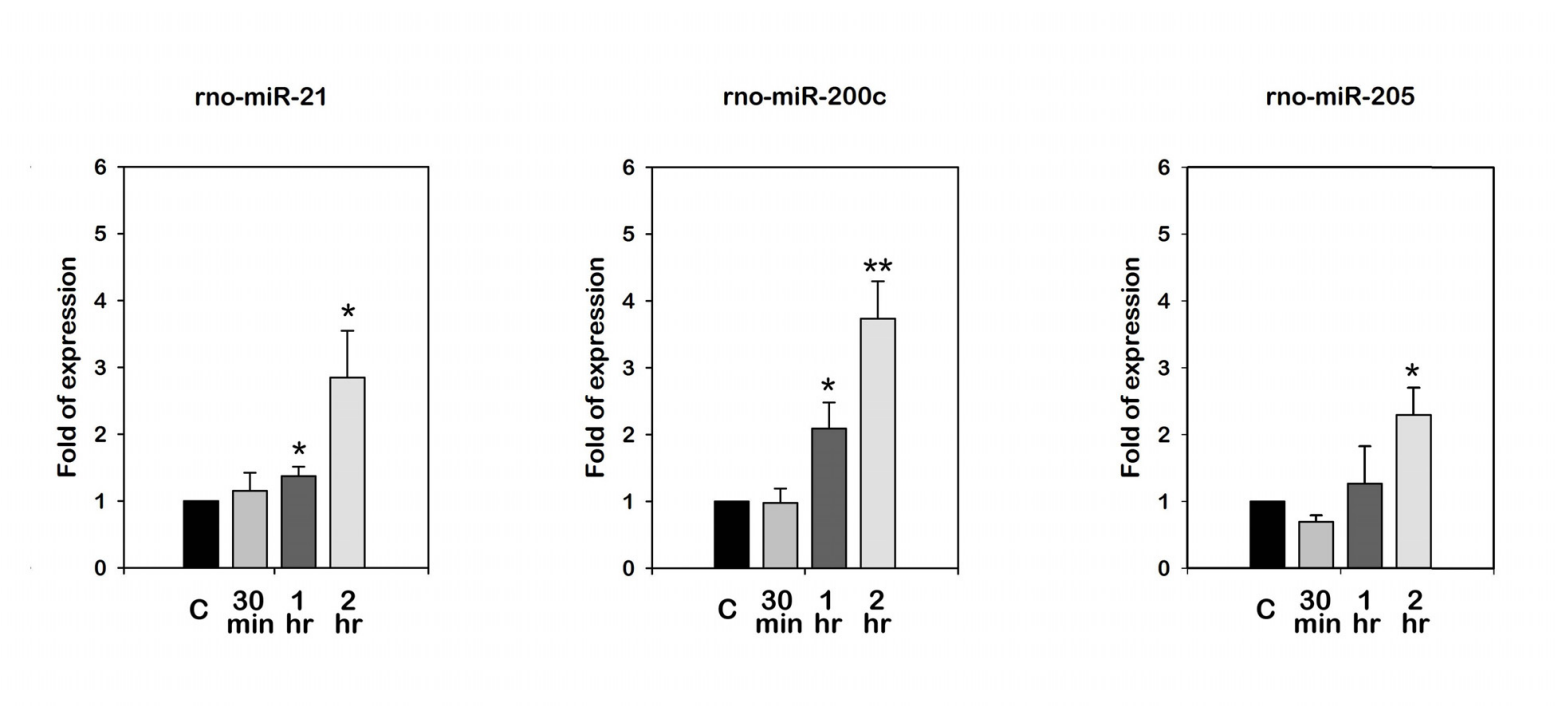
**Expression of miR-21, miR-200c, and miR-205 detected with real-time RT-PCR in the gracilis muscles following indicated ischemic times (30 min, 1 h, and 2 h) and reperfusion for 4 h**. Bars represent means ± standard deviation of 5 independent experiments; *, P < 0.05 vs. sham-operated control.

In the investigation of the differentially expressed miRNAs from the miRNA array experiments, there were only 3 miRNAs (miR-21, miR-200c, and miR-205) that showed an increased expression in the gracilis muscles after 4 h of ischemia and 4 h of reperfusion. We confirmed the microarray data by quantitative real-time RT-PCR to independently measure the relative expression of these 3 selected miRNAs in the samples of the experimental muscle. As shown in Figure 1, the 2-fold upregulation of miR-21 was detected after 4 h of ischemia and increased to a maximum of ~24 fold at 7 d after reperfusion. At 14 d, there was still a 7.5-fold increased expression of miR-21. The miR-200c expression was increased 3.2 fold and was detected 2 h after reperfusion following 4 h of ischemia. The expression of miR-200c reached its maximum level at 4 h and lasted for up to 8 h after ischemic injury. We did not detect an increased expression of miR-200c 1 d after reperfusion. A 6.4-fold upregulation of miR-205 was detected after 4 h of ischemia. It gradually decreased to 3.6-fold at 4 h after reperfusion. No significant difference in the expression of miR-205 was found at 8 h, 1 d, and 3 d after ischemic injury. Unexpectedly, we noted the 3.6-fold upregulation of miR-205 7 d later. This status persisted until 14 d after ischemic injury. In the experiment that we performed to identify the minimal ischemic time that would induce the expression of these 3 miRNAs, upregulation of miR-21 and miR-200c was noted after 4 h of reperfusion following 1 h and 2 h, but not 30 min of ischemia; in addition, upregulation of miR-205 was noted after 4 h of reperfusion following 2 h, but not 30 min or 1 h of ischemia (Figure 2).

### Finding the potential miRNA-regulated genes

**Figure 3:**
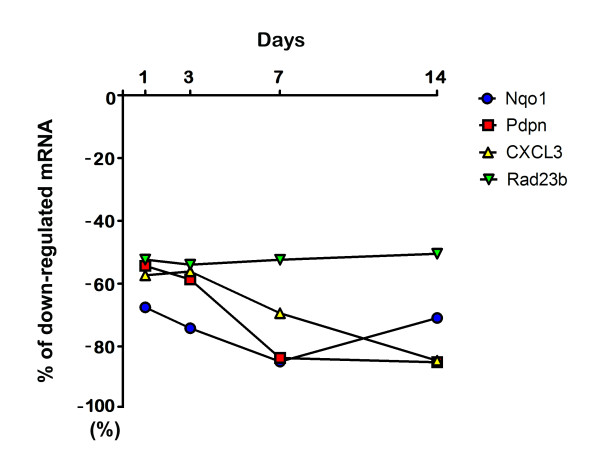
**The expression of down-regulated *Nqo1*, *Pdpn*, *CXCL3*, and *Rad23b*, which were the potential target genes of rno-miR-21 detected from the combined approach of the prediction algorithms miRanda and RNAhybrid, in the Whole Rat Genome 4 × 44 k oligo microarray experiments of the gracilis muscle after reperfusion for 1, 3, 7, and 14 d following 4 h of ischemia**.

Time-course studies provide information for the identification of miRNA-target mRNA pairs that could often be missed in a cross-sectional study when a single time point is used [26]. Thus, we examined mRNA expression patterns at the 4 indicated times (1, 3, 7, and 14 d after ischemic injury) based on the matched analysis of miRNA and mRNA expression data and considered an miRNA to be regulatory only if the upregulation in the expression profile of the miRNA and its predicted target mRNAs correlated. The miR-200c and miR-205 were not upregulated at all 4 indicated times. In addition, there was still lack of evidence regarding the effect on the persistent knockdown of mRNAs by an elevated miRNA. Therefore, we decided to only focus on the genes that were downregulated by miR-21, which showed a persistent expression throughout the experiment period at all 4 indicated times. The expression profiling using the Agilent rat 60-mer oligonucleotide microarrays showed there was a global pattern of differentially expressed genes at 1, 3, 7, and 14 d after ischemic injury. Expression profiling revealed that there were 1046 significantly downregulated gene transcripts in the experimental muscles (absolute expression changes were 2-fold or greater) at all 4 indicated times. In addition, the *in silico *prediction using the algorithms miRanda, PicTar, TargetScanS, MirTarget2, and RNAhybrid resulted in 957, 60, 159, 110, and 939 target genes of miR-21, respectively. The combination of the results of the computational prediction of target genes of miR-21 and the downregulation of genes in the whole genome expression array at 4 indicated times revealed no target gene on the basis of the prediction algorithms PicTar, TargetScanS, and MirTarget2; in contrast, when the prediction algorithms miRanda and RNAhybrid were applied, the same 4 target genes (*Nqo1*, *Pdpn*, *CXCL3*, and *Rad23b*) (Table 1) demonstrated a persistent downregulated status at 1, 3, 7, and 14 d (Figure 3). The miRNA array and the microarray data have been deposited in the Gene Expression Omnibus (accession number [GEO: GSE21423]).

**Table 1:** The potential regulated gene targets of rno-miR-21

Symbol	Full Name	Transcript ID	Gene Type
Nqo1	NAD(P)H dehydrogenase, quinone 1	ENSRNOT00000017174	Protein coding
Pdpn	podoplanin	ENSRNOT00000020316	Protein coding
Cxcl3	chemokine (C-X-C motif) ligand 3	ENSRNOT00000033592	Protein coding
Rad23b	RAD23 homolog B	ENSRNOT00000021629	Protein coding

Potential target genes of rno-miR-21 that were identified from downregulated mRNA in the experimental muscles in the whole genome expression array experiments after reperfusion for 1, 3, 7, and 14 d following 4 h of ischemia as well as from either the miRanda or RNAhybrid prediction algorithm.

## Discussion

In this study, we demonstrated that 3 miRNAs (miR-21, miR-200c, and miR-205) were significantly upregulated in a different pattern in the gracilis muscles following ischemic injury. Ischemia for only 1 h was sufficient to induce the expression of miR-21 and miR-200c during the reperfusion stage; and 2 h of ischemia were required to induce the expression of miR-205. Our data also showed that miR-200c and miR-205 were actively regulated after ischemia but their expression pattern changed after reperfusion, indicating their temporal expression during ischemic injury. In addition, with a yet to be determined mechanism, we noted a significant re-expression of miR-205 at 7 and 14 d after the injury. In contrast, the expression of miR-21 gradually increased to its maximum level at 7 d and persisted throughout the experiment for at least 14 d after the ischemic injury. This finding might imply an important role of miR-21 during ischemic injury in the muscle.

The overexpression of miR-21 has been shown to be in a number of medium-scale and high-scale profiling experiments that were designed for the detection of miRNAs that are dysregulated in cancer [27]. In addition, miR-21 has been reported to have anti-apoptotic properties in cancer cells [28,29]. It has been reported that miR-21 protects against the hydrogen peroxide-induced injury of cardiac myocytes via its target gene, the repressor gene programmed cell death 4 (*PDCD4*), and the AP-1 pathway [30]. The hydrogen peroxide-induced cardiac cell death and apoptosis were increased by a miR-21 inhibitor and was decreased by pre-miR-21 transfection [30]. In addition, a significant induction of miR-21 was noted in the heart following whole body heat-shock [19]. The injection of chemically synthesized exogenous miR-21 significantly reduced infarct size in the heart which was blocked with a miR-21 inhibitor [19]. In an investigation of rat hearts at 6 h after acute myocardial injury, miRNA signatures in the early phase revealed that, among multiple aberrantly expressed miRNAs, miR-21 was significantly downregulated in infarcted areas, but was upregulated in border areas [31]. Remarkably, the downregulation of miR-21 in infarcted areas was inhibited by ischemic preconditioning, a known cardio-protective method. In addition, adenoviral overexpression of miR-21 had a protective effect on myocardial infarction by decreasing the infarct size by 29% at 24 h [31]. In this study, we found 4 potential target genes (*Nqo1*, *Pdpn*, *CXCL3*, and *Rad23b*) of miR-21 during skeletal muscle ischemic injury. The database of experimentally validated miRNA target genes, MiRecords [32], lists 26 validated target genes of miR-21 in humans (*TPM1*, *CDK6*, *TIMP3*, *PDCD4*, *SERPINB5*, *NFIB*, *CDKN1A*, *FAS*, *FAM3C*, *HIPK3*, *PRRG4*, *ACTA2*, *BTG2*, *BMPR2*, *SESN1*, *IL6R*, *SOCS5*, *GLCCI1*, *APAF1*, *SLC16A10*, *SGK3*, *RP2*, *CFL2*, *RECK*, *MTAP*, *SOX5*) and only 2 validated target genes in the rat (*ITGB1*, *Tagln*). Interestingly, the 4 genes that we identified in this study did not match with the already validated target genes in humans or rats. Among these 4 identified target genes, podoplanin (Pdpn) is a transmembrane glycoprotein and is expressed in many normal human tissues including the skeletal muscle [33]. Pdpn is widely used as a specific marker for lymphatic endothelial cells and lymphangiogenesis in many species because it is expressed on lymphatic but not on blood vessel endothelium [33]. Deficiency of Pdpn results in congenital lymphedema and impaired lymphatic vascular patterning [34]. With the postfix L (for ligand) or postfix R (for receptor), CXCL and CXCR are used as ligands and receptors of the CXC chemokine family which exhibits both angiogenic and angiostatic properties [35]. While chemokines can exert a pro-angiogenic effect via recruitment of inflammatory cells, *CXCL3 *is induced in the human perihematomal tissue [36] and can mediate angiogenesis in the absence of preceding inflammation [35]. The nucleotide excision repair (NER) protein, Rad23b, was found to be downregulated in hypoxic cancer cells [37]. Hypoxia can also promote genetic instability by affecting the DNA repair capacity, including NER which primarily focuses on helix-distorting injuries [38]. The downregulation of Rad23b in hypoxic cancer cells could be partially reversed by antisense inhibition of miR-373 [37], indicating a key role of miR-373 in modulating the basal expression of Rad23b. The effect of anti-miR-373 activity in normoxia is more profound than in hypoxia; thus, it has been suggested that there might be other mechanisms that regulate the expression of Rad23b [37]. In reviewing the literature, no direct linkage between these 4 identified target genes and the muscle ischemic injury was found. Only the detoxification enzyme NAD(P)H: quinone oxidoreductase 1 (Nqo1) could be correlated to the ischemic injury in renal tissue [39] and primary cultures of rat cortex [40], but with different outcomes regarding the cytoprotective effect. As a detoxification enzyme Nqo1 catalyzes the 2-electron reduction of quinoid compounds to the readily excreted hydroquinones, thereby preventing the generation of reactive oxygen species and protecting cells against oxidative damage [41]. The expression of the *Nqo1 *gene is primarily regulated via antioxidant response element sequences in the promoter region. Reoxygenation-specific activation of the antioxidant transcription factor Nrf2 mediates the cytoprotective gene expression during ischemia-reperfusion injury [42]. However, some authors suggested a deteriorating rather than a protective factor of Nqo1 in the progression of neuronal cell death, as inhibition of Nqo1 by various inhibitors protects against neuronal damage in vitro and following cerebral ischaemia in vivo [40]. Because bioinformatic analysis has indicated that miRNAs frequently interact with transcription factors in feedback and feedforward loops to regulate their target genes [43,44]; therefore, further experiments are required to elucidate the function and role of these potential target genes and the upregulated miR-21, as well as miR-200c and miR-205, in ischemic injury.

The pneumatic tourniquet is frequently used in surgery to acquire a bloodless operative field. To prevent the ischemia-reperfusion injury, the maximal allowable ischemic time of the pneumatic tourniquet is 2 h [45,46] Although minor histological changes in muscle started to appear after about 35-40 minutes, there was no clinical evidence of irreversible muscle damage within 2 h [45-47]; this is also the reason why most of the article studying the ischemic injury of skeletal muscle would choose the ischemic time at 4 h or a longer ischemic time. Whether there is a different miRNAs expression profile after a shorter time of ischemia such as 1 h or 2 h is unknown, but if these miRNAs are not up-regulated in a longer hour ischemia, the investigation of these targets may be devoid of clinical importance or attention. Therefore, in this study, we were not intended to profile the miRNAs expression with a miRNA array at a shorter time, like 2 h, of ischemia. However, as shown in the Figure 2, in the investigation of minimal ischemic time that would induce the expression of these 3 miRNAs, we had demonstrated that 1 h of ischemia was able to increase the expression of miR-21 and miR-200c and 2 h of ischemia would increase the expression of these three miRNAs, implying the epigenetic regulation could be induced by a shorter time of ischemia before a remarked pathophysiologic change could be observed by a longer time of ischemia.

Bioinformatic algorithms remain the principal means of predicting targets of specific miRNAs. These algorithms take into account numerous parameters that influence miRNA/target interactions, including seed match (complementarity), 3'-UTR seed match context, seed match conservation, favorability of free energy binding, AU content, and binding site accessibility [48]. To identify miRNAs that regulate mRNAs, one needs to co-analyze the changes in miRNA and mRNA expressions. The investigation of the mRNA:miRNA association monitors the miRNA and mRNA expression on a genome-wide basis and provides an analytical approach to reveal the target genes of the miRNA [49,50]. However, some limitations still remain for the combined approach method that we described here. First, it might be overly simplistic to correlate miRNAs and their predicted targets primarily on the basis of the number of consensus sites in the 3'UTR because an exact match to the sequence of the seed region is not required. For example, miRanda typically produces more potential targets than other programs, but a large number of false targets would seriously limit the value of the output information [51]. In contrast, some available programs with stricter criteria, e.g., the prediction algorithms PicTar, TargetScanS, and MirTarget2 have only partially overlapping predicted targets for the same miRNA and produce smaller data sets than miRanda [52]. In this present study, in order to reduce the noise from the calculation of the correlation between miRNA and mRNAs (based on the time-course expression values), the inclusion of downregulated genes at all 4 time points might have been too strict to acquire potential target genes. In addition, it might have produced a smaller number of predicted targets after correlation with the stricter prediction algorithm considering the accuracy and reproducibility of the whole genome array. For example, no target genes were found for the combination with the prediction algorithms PicTar, TargetScanS, and MirTarget2. Due to the differences among databases and because there is no clear superior method, a further gain-of-function or loss-of function experiment would be helpful to elucidate the role of the identified target genes and that of each associated miRNA. Furthermore, it has been reported that more than a third of those translationally repressed target genes always displayed detectable mRNA destabilization [14]. However, the extent of miRNA function in animal cells has largely been studied by mRNA microarray profiling assuming that miRNA function leads to reduced mRNA levels, which may not be always the case. There might be some unidentified target genes that are repressed only in the translation process but have not been subjected to mRNA degradation.

## Conclusions

This study has profiled an increased expression of miR-21, miR-200c, and miR-205 in the gracilis muscle following ischemic injury and identified four potential target genes (*Nqo1*, *Pdpn*, *CXCL3*, and *Rad23b*) of the miR-21 by using different prediction algorithms and monitoring the expression of miRNA and mRNA at different time point on a genome-wide basis. Although the exact roles of these upregulated miRNAs following ischemic injury remains to be elucidated, this study provides a novel insight into the epigenetic regulation in the skeletal muscle following ischemic injury.

## List of abbreviations

CXCL3: chemokine (C-X-C motif) ligand 3; miRNA: microRNA; Nqo1: NAD(P)H: quinone oxidoreductase 1; Pdpn: podoplanin; Rad23b: RAD23 homolog B.

## Competing interests

The authors declare that they have no competing interests.

## Authors' contributions

CHH was responsible for the design and coordination of the data acquisition and analysis, search for target genes via the computational algorithm, interpretation of the data, and the writing of the manuscript. JCJ and CJW participated in the real-time RT-PCR experiment. SFJ and PCL participated by providing and coordinating the resources. THL contributed to the animal surgery and acquisition of the study specimens. CSR participated in the analysis and interpretation of the data. YCC and CJL were involved in the acquisition of the miRNA array and whole genome expression data. All authors read and approved the final manuscript.

## Pre-publication history

The pre-publication history for this paper can be accessed here:

http://www.biomedcentral.com/1471-2474/11/123/prepub
